# Food Security Status and Barriers to Fruit and Vegetable Consumption in Two Economically Deprived Communities of Oakland, California, 2013–2014

**DOI:** 10.5888/pcd13.150402

**Published:** 2016-02-11

**Authors:** Kim Mook, Barbara A. Laraia, Vanessa M. Oddo, Jessica C. Jones-Smith

**Affiliations:** Author Affiliations: Kim Mook, Vanessa M. Oddo, Jessica C. Jones-Smith, Johns Hopkins University Bloomberg School of Public Health, Baltimore, Maryland; Barbara A. Laraia, University of California, Berkeley School of Public Health, Berkeley, California.

## Abstract

**Introduction:**

Food security status may moderate how people perceive barriers to fruit and vegetable consumption. This study aimed to 1) describe the association between fruit and vegetable consumption and microbarriers and mezzobarriers to consumption, and 2) test whether these associations differ by food security status.

**Methods:**

We surveyed adults (n = 531) living in 2 economically deprived communities in Oakland, California, in 2013 and 2014. Multivariate linear regression assessed associations between microbarriers (taste, cost, busyness) and mezzobarriers (produce selection, quality, and purchase ease) and fruit and vegetable consumption, derived from a 26-item dietary screener. Interactions were tested by food security status.

**Results:**

Respondents consumed a mean 2.4 (standard deviation, 1.5) servings of fruits and vegetables daily; 39% of the sample was food insecure. Being too busy to prepare healthy foods was associated with reduced fruit and vegetable consumption (β_busyness_ = −0.40; 95% confidence interval [CI], −0.52 to −0.28) among all respondents. Food security moderated the relationship between fruit and vegetable consumption and taste, cost, and perceived ease of purchase of healthy foods. Among the food secure, disliking healthy food taste (β_taste_ = −0.38; 95% CI, −0.60 to −0.15) and cost (β_cost_ = −0.29; 95% CI, −0.44 to −0.15) concerns were associated with lower consumptions of fruits and vegetables. Mezzobarriers were not significantly associated with consumption in either group.

**Conclusion:**

Perceived time constraints influenced fruit and vegetable consumption. Taste and cost influenced fruit and vegetable consumption among the food secure and may need to be considered when interpreting analyses that describe dietary intake and designing diet-related interventions.

## Introduction

Poor diet, or consumption of foods high in fat, salt, and cholesterol, is a leading risk factor for obesity, heart disease, and cancer (1,2). Diets rich in high-nutrient foods, such as fruits and vegetables, can promote health, prevent obesity, and lower risk for heart disease, stroke, type 2 diabetes, and cancer (3,4). Fruit and vegetable consumption in America is low; only 18% of people meet the dietary guidelines for fruits, and 13% meet guidelines for vegetables (5). Groups at greater risk than others for low fruit and vegetable consumption are young adults (6–8), men (6,7,9,10), African Americans (7,8,11,12), and those of low income or educational status (8,13,14).

Studies investigating fruit and vegetable consumption have identified several factors associated with consumption. The most common factors are taste preferences (9,10,14–17), food preparation time (9,15,18), cost (9,18), and access (14,15,19). These factors have been investigated among diverse populations, including those of different age, sex, socioeconomic status, and race/ethnicity. Food environment, including food cost and availability, can also create barriers to healthy food that differentially affect low-income households (14,15,19,20).

Most studies investigating barriers to fruit and vegetable consumption control for socioeconomic variables; few studies consider food insecurity. Food-insecure households are commonly defined as having “limited or uncertain access to adequate food” (21). Access is often defined as access to sources of healthy food, as measured by distance to or the number of stores in an area; individual-level resources, such as family income; or neighborhood-level resources, such as availability of public transportation (22). Although food insecurity and poverty are highly correlated, these categories are not synonymous (23). Socioeconomic variables do not consider whether household resources negatively affect food consumption and purchase decisions. Food-insecure families may approach food decisions differently than do food-secure families, regardless of income (24). Controlled for income, food insecurity is associated with poor nutrition and diet (23,25,26), poor health (27), and higher rates of female obesity (28,29). Other factors may influence dietary habits of food-insecure populations, and families may use various mechanisms to cope with lack of resources (24).

A better understanding of barriers to healthy food consumption in food-secure and food-insecure populations would facilitate the creation of more targeted interventions to improve diet and health. The objective of this study was to investigate whether the relationship between barriers to healthy food consumption and reported consumption rates differ by food-security status. We hypothesized that food quality and taste preferences would be more strongly associated with food choices in food-secure populations than in food-insecure populations because food-secure populations may have more opportunities to align food quality and taste choices with their preferences. Conversely, we also hypothesized that cost constraints, selection, and ease of purchase would be more strongly associated with food choice in food-insecure populations than in food-secure populations. Furthermore, we hypothesized that busyness, as it relates to food preparation time, would be associated with healthy eating in both populations.

## Methods

This analysis used cross-sectional data from a parent study that assessed the impact of new supermarkets in economically deprived neighborhoods on the diets of neighborhood residents in Oakland, California, in 2013 and 2014. All respondents resided in 2 neighborhoods where the FoodsCo chain planned to open new supermarkets. We obtained participant information from a commercial database and sent recruitment materials to all residents (n = 10,792) in contiguous, economically deprived (determined by a neighborhood deprivation index [30]) census tracts within an approximate 2-mile radius of each planned supermarket. Consenting residents (n = 636) completed the survey by mail or online. The analytic sample consisted of 531 respondents after elimination of nonsampled addresses (n = 8) and surveys missing data for the dependent variable (n = 51), the independent variable (n = 14), food security status (n = 14), and confounding variables (n = 18). When a respondent marked more than one response to a question, one response was randomly selected for analysis. The Johns Hopkins Institutional Review Board approved the study protocol, including an unsigned, passive consent form.

Using the Glass and McAtee framework as a guiding theoretical model, we examined both microbarriers (ie, individual, group, family, or network characteristics) and mezzobarriers (ie, community, work-site, or school characteristics) (31). We used this framework for several reasons: 1) it aims to understand what differentially places people at risk for risks, 2) it adapts the stream-of-causation metaphor and describes how social factors can be “risk regulators” of behaviors, 3) its conceptualization overlaps with the concept of social and economic factors (such as food insecurity) as effect-measure modifiers of the relationship between an exposure and an outcome, and 4) unlike models with typical conceptual categories (eg, household-level), it details the interrelationships among nested levels, rather than just within levels.

Data for the dependent variable, average daily servings of fruits and vegetables, were collected by using a 26-item dietary screener (32). Consumption during one month was self-reported on a 10-item scale ranging from “never” to 6 or more times daily. The questions on fruit and vegetable consumption have acceptable agreement with a 24-hour recall for women (*R* = 0.5–0.8) and men (*R* = 0.6–0.7). Average monthly consumption was converted to daily frequency, as suggested by the Centers for Disease Control and Prevention (33). Reported consumption rates of fruit (fresh, frozen, or canned), green leafy vegetables, and other vegetables were summed to produce a continuous variable describing average daily servings of fruits and vegetables.

The independent variables were derived from 6 survey questions on barriers to healthy food consumption. Questions on the availability of healthy foods (34) and food attitudes (35) were derived from published scales. An exploratory factor analysis of the 6 barrier variables produced 2 categories: 1) taste, cost, and preparation time (Cronbach α = 0.63) and 2) selection, quality, and availability (Cronbach α = 0.90). Taste, cost, and time constraints were conceptualized as microbarriers to healthy food consumption. Microbarriers are factors at the lowest-level — ie, groups, individuals, or social networks (31). For example, participants were asked the degree to which they agreed or disagreed with the statement, “I don’t think healthy foods taste good.” Mezzobarriers are factors at higher levels — ie, work-sites, schools, and communities (31). For example, participants were asked about the selection of fresh fruits and vegetables in or near their neighborhood. Response options for both categories of questions were the following: strongly disagree, disagree, neutral, agree, and strongly agree. Mezzobarrier responses were recoded in the opposite direction to be consistent with microbarrier responses; higher scores indicate stronger agreement that a factor is a barrier. All barrier variables were ordinal, based on Likert scale, centered on neutral to allow for interpretation with interaction terms (−2 = strongly disagree, −1 = disagree, 0 = neutral, 1 = agree, and 2 = strongly agree), and modeled as separate independent variables in regression analysis.

Data on food security status were collected by using the US Department of Agriculture’s 6-item short-form food security scale (21). Food security status was categorized as food secure (raw score 0–1) or food insecure (raw score 2–6) and analyzed as a dichotomous variable (21). 

Education (no degree; high school degree or general educational development; trade school, occupational, technical or vocational certificate; some college; college degree or higher), annual household income (<$10,000; $10,000–$24,999; $25,000–$49,999; $50,000–$79,999; ≥$80,000), and age (continuous variable) were self-reported. Body mass index (BMI) was calculated by using self-reported height and weight.

### Statistical analysis

Linear regression was used to investigate the relationship between daily servings of fruits and vegetables and each perceived barrier. To determine which covariates to include in analysis, variable relationships were modeled in directed acyclic graphs (36). We used DAGitty software (37) to determine the minimally sufficient set of confounders needed in each model. Food security was hypothesized as an effect-measure modifier between each barrier and fruit and vegetable consumption; if food security was not an effect-measure modifier, it was conceptualized as a potential confounder. Model building began with the full set of hypothesized confounders, including age, income, and education level (Model 1). Each model was then tested for potential effect modification by food security status and the main independent variable of interest; all significant interactions were retained. This model was then tested for interactions between each confounder and food security status or among the confounders. No other interactions were found between any other confounder and the independent variables or among the confounders (36). Models for taste, cost, and ease of purchase included the significant interaction term between food security and the independent variable.

Variable specifications were investigated for age, income, and education. Disjoint indicator variables were specified for age, income, and education, and the degree the outcome increased for each category was assessed. Based on these assessments, income was modeled as ordinal, age was modeled continuously with a squared age term, and education was collapsed into 2 categories: 1) college degree or 2) less than a college degree. We performed a separate analysis (Model 2) to investigate only the minimally sufficient set of confounders necessary for each independent variable.

Residual-versus-fitted plots were visually inspected for final models and showed evidence of heteroskedasticity in model error terms. Several data transformations were investigated but none improved residual diagnostics, so Huber–White robust standard errors were used to produce heteroskedastic-robust standard errors (38). Significance levels were set at .05 for main effects and .10 for interaction terms (assessed by *F* tests) (36), and all analysis was conducted in Stata version 13.1 (StataCorp) in 2014.

## Results

The mean age of respondents was 47.9 years and mean BMI was 29.2; 73.6% were women (Table 1). Approximately 40% (208 of 531) of the sample was food insecure. A higher percentage of food-insecure respondents (28.4%) than food-secure respondents (5.6%) had an annual household income of less than $10,000. Similarly, 3.8% of food-insecure respondents and 38.4% of food-secure respondents reported an income of $80,000 or more. Among food-insecure respondents, 59.6% received a college degree, whereas 84.5% of food-secure respondents received one.

**Table 1:** Characteristics of Respondents in Survey on Fruit and Vegetable Consumption and Food Insecurity in Two Economically Deprived Neighborhoods of Oakland, California, by Food Security Status, 2013–2014^a^

Variable	Food Secure (n = 323)	Food Insecure (n = 208)	Total (N = 531)
**Age, mean (SD)**	49.7 (11.5)	45.2 (12.5)	47.9 (12.1)
**Body mass index, mean (SD)**	28.0 (6.5)	30.9 (8.6)	29.2 (7.5)
**Sex**			
Male	96 (29.7)	41 (19.7)	137 (25.8)
Female	225 (69.7)	166 (79.8)	391 (73.6)
**Annual household income, $**			
<10,000	18 (5.6)	59 (28.4)	77 (14.5)
10,000–$4,999	35 (10.8)	74 (35.6)	109 (20.5)
25,000–49,999	70 (21.7)	52 (25.0)	122 (23.0)
50,000–79,999	76 (23.5)	15 (7.2)	91 (17.1)
≥80,000	124 (38.4)	8 (3.8)	132 (24.9)
**Education**			
Less than college degree	50 (15.5)	84 (40.4)	134 (25.2)
College degree or more	273 (84.5)	124 (59.6)	397 (74.8)
**Self-reported health status**			
Poor	1 (0.3)	7 (3.4)	8 (1.5)
Fair	30 (9.3)	42 (20.2)	72 (13.6)
Good	101 (31.3)	88 (42.3)	189 (35.6)
Very good	132 (40.9)	60 (28.8)	192 (36.2)
Excellent	59 (18.3)	11 (5.3)	70 (13.2)
**Access to car**			
Yes	17 (5.3)	43 (20.7)	60 (11.3)
No	305 (94.4)	164 (78.8)	469 (88.3)
**Race/ethnicity**			
Black	142 (44.0)	130 (62.5)	272 (51.2)
Hispanic	47 (14.6)	53 (25.5)	100 (18.8)
White	114 (35.3)	27 (13.0)	141 (26.6)
Asian	28 (8.7)	8 (3.8)	36 (6.8)
Native American	1 (0.3)	2 (1.0)	3 (0.6)
Alaska Native	1 (0.3)	0 (0.0)	1 (0.2)
Pacific Islander	1 (0.3)	1 (0.5)	2 (0.4)
Other race/ethnicity	8 (2.5)	10 (4.8)	18 (3.4)

Abbreviation: SD, standard deviation.

a All values are number (percentage) unless otherwise indicated.

On average, respondents consumed 2.4 servings of fruit and vegetables daily (standard deviation [SD], 1.5) (Table 2). Food-secure respondents consumed 2.7 daily servings (SD, 1.5), and food-insecure respondents consumed 1.9 daily servings (SD, 1.4). A higher percentage of food-insecure respondents than food-secure respondents agreed or strongly agreed they were too busy to prepare healthy foods (21.1% vs 14.2%), that healthy foods cost too much (43.7% vs 13.3%), and that they did not like the taste of healthy foods (12.0% vs 5.6%).

**Table 2:** Results of Survey on Fruit and Vegetable Consumption and Food Insecurity in Two Economically Deprived Neighborhoods of Oakland, California, by Food Security Status, 2013–2014^a^

Measure	Food Secure (n = 323)	Food Insecure (n = 208)	Total (N = 531)
**Daily servings of fruits and vegetables, mean (SD)**	2.7 (1.5)	1.9 (1.4)	2.4 (1.5)
**Microbarriers to healthy food consumption**			
I don’t think healthy foods taste good			
Strongly disagree	179 (55.4)	67 (32.2)	246 (46.3)
Disagree	107 (33.1)	86 (41.3)	193 (36.3)
Neutral	19 (5.9)	30 (14.4)	49 (9.2)
Agree	12 (3.7)	14 (6.7)	26 (4.9)
Strongly agree	6 (1.9)	11 (5.3)	17 (3.2)
It costs too much for me to eat healthy foods			
Strongly disagree	121 (37.5)	18 (8.7)	139 (26.2)
Disagree	108 (33.4)	47 (22.6)	155 (29.2)
Neutral	51 (15.8)	52 (25.0)	103 (19.4)
Agree	34 (10.5)	62 (29.8)	96 (18.1)
Strongly agree	9 (2.8)	29 (13.9)	38 (7.2)
I’m too busy to take the time to prepare healthy foods			
Strongly disagree	99 (30.7)	30 (14.4)	129 (24.3)
Disagree	131 (40.6)	87 (41.8)	218 (41.1)
Neutral	47 (14.6)	47 (22.6)	94 (17.7)
Agree	44 (13.6)	34 (16.3)	78 (14.7)
Strongly agree	2 (0.6)	10 (4.8)	12 (2.3)
**Mezzobarriers to healthy food consumption**			
A large selection of fresh fruits and vegetables are available in or near my neighborhood			
Strongly agree	53 (16.4)	29 (13.9)	82 (15.4)
Agree	112 (34.7)	70 (33.7)	182 (34.3)
Neutral	47 (14.6)	40 (19.2)	87 (16.4)
Disagree	54 (16.7)	42 (20.2)	96 (18.1)
Strongly disagree	57 (17.6)	27 (13.0)	84 (15.8)
It is easy to purchase fresh fruits and vegetables in or near my neighborhood			
Strongly agree	55 (17.0)	23 (11.1)	78 (14.7)
Agree	125 (38.7)	82 (39.4)	207 (39.0)
Neutral	34 (10.5)	36 (17.3)	70 (13.2)
Disagree	56 (17.3)	39 (18.8)	95 (17.9)
Strongly disagree	53 (16.4)	28 (13.5)	81 (15.3)
The fresh fruits and vegetables in or near my neighborhood are of high quality			
Strongly agree	35 (10.8)	18 (8.7)	53 (10.0)
Agree	100 (31.0)	44 (21.2)	144 (27.1)
Neutral	75 (23.2)	69 (33.2)	144 (27.1)
Disagree	63 (19.5)	43 (20.7)	106 (20.0)
Strongly disagree	50 (15.5)	34 (16.3)	84 (15.8)

Abreviation: SD, standard deviation.

a All values are number (percentage) unless otherwise indicated.

**Figure 1:**
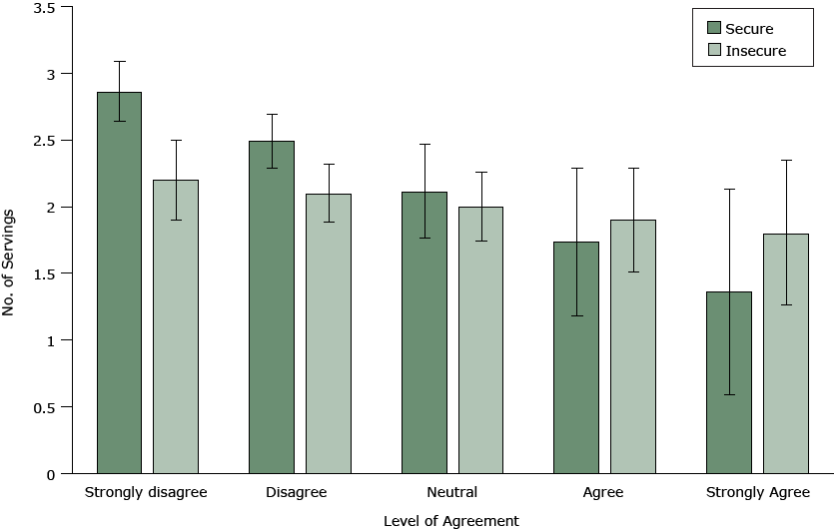
The relationship between the degree of agreement with the statement, “I don’t think healthy foods taste good” and the number of average daily servings of fruits and vegetables, by food security status, Oakland, California, 2013–2014. **Table:** 

Food security modified the relationship between distaste for healthy food and fruit and vegetable consumption (taste × food insecurity [FIS]: *P* = .05). Among food-secure respondents, greater distaste for healthy foods was associated with lower fruit and vegetable consumption (β_taste_ = −0.38 servings; 95% confidence interval [CI], −0.60 to −0.15; *P* < .001). Among food-insecure respondents, distaste for healthy food was associated with a nonsignificant 0.10 decrease in daily servings of fruit and vegetables (β_taste_ + [β_taste_ × FIS] = −0.10; 95% CI, −0.28 to 0.08; *P* = .26) (Table 3 and Figure 1).

**Table 3:** Linear Regression Estimates for the Associations Between Barriers to Healthy Food Consumption, Survey on Fruit and Vegetable Consumption and Food Insecurity in Two Economically Deprived Neighborhoods of Oakland, California, by Food Security Status, 2013–2014

Barriers	Model 1^a^ (N = 531)	
	β (95% CI)	*P* Value
**Microbarriers**		
I don’t think healthy foods taste good	−0.38 (−0.60 to −0.15)	<.001
Food insecurity	−0.11 (−0.57 to 0.34)	.62
Taste × food insecurity	0.27 (−0.0013 to 0.55)	.05
It costs too much for me to eat healthy foods	−0.29 (−0.44 to −0.15)	<.001
Food insecurity	−0.21 (−0.54 to 0.12)	.20
Cost × food insecurity	0.23 (0.001 to 0.46)	.05
I’m too busy to take the time to prepare healthy foods	−0.40 (−0.52 to −0.28)	<.001
Food insecurity	−0.25 (−0.56 to 0.06)	.11
**Mezzobarriers**		
The fresh fruits and vegetables in or near my neighborhood are of high quality	−0.08 (−0.12 to 0.10)	.88
Food insecurity	−0.44 (−0.77 to −0.12)	.01
A large selection of fresh fruits and vegetables are available in or near my neighborhood	0.07 (−0.032 to 0.16)	.19
Food insecurity	−0.47 (−0.79 to −0.14)	.004
It is easy to purchase fresh fruits and vegetables in or near my neighborhood	0.11 (−0.019 to 0.24)	.09
Food insecurity	−0.51 (−0.84 to −0.18)	.002
Purchase × food insecurity	−0.21 (−0.41 to −0.02)	.03

Abbreviation: CI, confidence interval.

a Estimated using linear regression controlling for age, income, education, and food security.

Survey Response	No. of Average Daily Servings (95% Confidence Interval)	
	Food Secure	Food Insecure
Strongly disagree	2.86 (2.64–3.09)	2.20 (1.90–2.50)
Disagree	2.49 (2.29–2.69)	2.10 (1.88–2.32)
Neutral	2.11 (1.76–2.47)	2.00 (1.74–2.26)
Agree	1.74 (1.18–2.29)	1.90 (1.51–2.29)
Strongly agree	1.36 (0.59–2.13)	1.80 (1.25–2.34)

**Figure 2:**
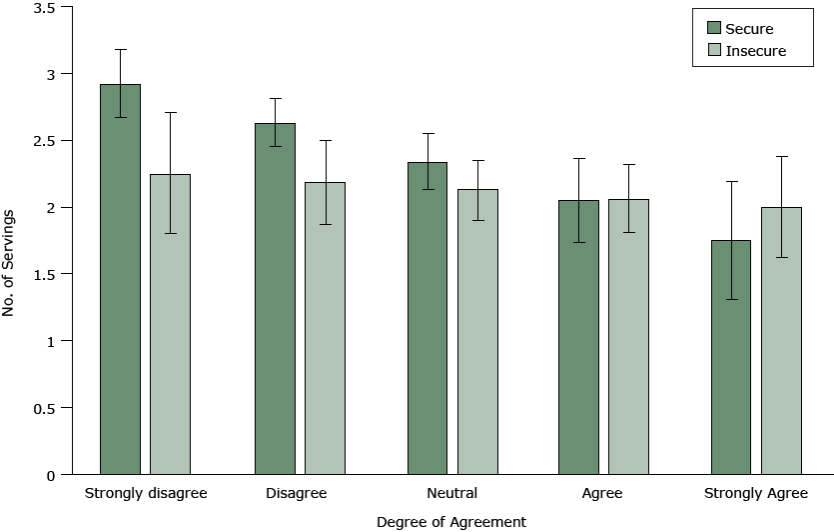
The relationship between the degree of agreement with the statement, “It costs too much for me to eat healthy foods” and the number of average daily servings of fruits and vegetables, by food security status, Oakland, California, 2013–2014. **Table:** 

Food security was also a significant modifier of the relationship between cost and healthy food consumption (cost × FIS: *P* = .049). Among the food secure, cost concerns were associated with a significant decrease in daily servings of fruits and vegetables (β_cost_ = −0.29; 95% CI, −0.44 to −0.15; *P* < .001) (Table 3). Among the food insecure, cost concerns were associated with a nonsignificant 0.06 decrease in daily servings of healthy foods (β_cost_ + [β_cost_ × FIS] = −0.06; 95% CI, −0.24 to 0.12; *P *= .50) (Table 3 and Figure 2).

Likert scale	Average Daily Servings of Fruits and Vegetables (95% Confidence Interval)	
	Food Secure	Food Insecure
Strongly disagree	2.92 (2.67–3.18)	2.25 (1.80–2.71)
Disagree	2.63 (2.45–2.81)	2.19 (1.87–2.50)
Neutral	2.34 (2.13–2.55)	2.13 (1.90–2.35)
Agree	2.05 (1.73–2.36)	2.06 (1.81–2.32)
Strongly agree	1.75 (1.31–2.19)	2.00 (1.62–2.38)

Food security status did not modify the relationship between busyness and consumption (busyness × FIS: *P* = .25). Busyness was associated with significantly 0.40 fewer (95% CI, −0.52 to −0.28;* P* < .001) daily servings of fruits and vegetables among food-secure and food-insecure respondents (Table 3).

Among mezzobarriers, food security was a modifier only on ease of purchase and consumption (purchase × FIS: *P* = .03), however, we found no significant association between ease of purchase and consumption among food-secure or food-insecure respondents. No mezzobarrier had a significant association with healthy food consumption among food-secure or food-insecure respondents (Table 3). We found no significant differences between Model 1 and Model 2 (Table 4).

**Table 4:** Linear Regression Estimates for the Associations Between Barriers to Healthy Food Consumption and Fruit and Vegetable Intake, Controlling for Only the Minimally Sufficient Set of Confounders, Survey on Fruit and Vegetable Consumption and Food Insecurity in Two Economically Deprived Neighborhoods of Oakland, California, by Food Security Status, 2013–2014^a^

Barriers	Model 2 (N = 531)	
	β (95% CI)	*P* Value
**Microbarriers**		
I don’t think healthy foods taste good	−0.42 (−0.64 to −0.21)	<.001
Food insecurity	−0.26 (−0.68 to 0.15)	.22
Taste × food insecurity	0.30 (0.02 to 0.57)	.03
It costs too much for me to eat healthy foods	−0.29 (−0.44 to −0.15)	<.001
Food insecurity	−0.23 (−0.55 to 0.10)	.17
Cost × food insecurity	0.22 (−0.005 to 0.45)	.05
I’m too busy to take the time to prepare healthy foods	−0.39 (−0.51 to −0.28)	<.001
Food insecurity	−0.61(−0.86 to −0.36)	<.001
**Mezzobarriers**		
The fresh fruits and vegetables in or near my neighborhood are of high quality	−0.08 (−0.12 to 0.10)	.88
Food insecurity	−0.44 (−0.77 to −0.12)	.01
A large selection of fresh fruits and vegetables are available in or near my neighborhood	0.07 (−0.03 to 0.16)	.19
Food insecurity	−0.47 (−0.79 to −0.14)	.004
It is easy to purchase fresh fruits and vegetables in or near my neighborhood	0.11 (−0.02 to 0.24)	.09
Food insecurity	−0.51 (−0.84 to −0.18)	.002
Purchase × food insecurity	−0.21 (−0.41 to −0.02)	.03

Abbreviation: CI, confidence interval

a Estimated using linear regression and controls for minimally sufficient set of confounders according to our directed acyclic graph, which were age and food security for taste; income, age, and food security for cost; nothing for preparation time; and income, age, food security, and education for all mezzobarriers. The model for “busyness” did not consider food security a theoretical confounder or was not found to be an effect-measure modifier. However, food security status was controlled for to report on the effects of food security on all independent variables.

## Discussion

To our knowledge, ours is the first study to test whether established barriers to reported fruit and vegetable consumption are similar among food-insecure people and food-secure people in an urban, economically deprived sample. Similar to most Americans, the average respondent in our study did not meet recommendations for fruit and vegetable intake. Food-insecure respondents reported 1.9 daily servings, and food-secure respondents reported 2.7 servings; the recommended consumption is 5.5 servings (39). We found important differences by food security status in the relationship between perceived barriers to consumption and self-reported fruit and vegetable consumption, after controlling for income. Our findings are consistent with those of Edin et al (24), who reported low-income food-insecure populations use various mechanisms to cope with food shortfalls, such as restricting food consumption (ie, skipping meals) and relying on inexpensive starches. Among our sample, distaste for healthy foods was associated with decreased fruit and vegetable consumption only among food-secure respondents. Busyness was associated with lower consumption among both food-secure and food-insecure respondents. In contrast to our hypothesis, perceiving that healthy foods cost too much was associated with lower consumption only among food-secure respondents. These results suggest that public health researchers and practitioners should consider food security status, regardless of income, when studying and intervening on healthy food consumption.

**Microbarriers.** Among food-secure respondents, cost, taste, and busyness were significantly associated with fruit and vegetable consumption. These results are consistent with the results of other studies that found associations between preparation time, taste, and cost with daily consumption of fruits and vegetables (5–15,17). Interventions that make healthy foods more appealing, easy to prepare, and more cost-efficient may be appropriate among food-secure populations.

We found consistent associations among food-secure respondents between healthy food consumption and microbarriers. Food-insecure respondents reported microbarriers more frequently than did food-secure respondents; they were more likely to report cost, preparation time, and taste as barriers to healthy food consumption. While microbarriers were perceived more frequently among the food insecure, they were not consistently associated with fruit and vegetable consumption.

Fruit and vegetable consumption among the food insecure was associated with busyness but not taste or cost. The lack of association with taste preferences is consistent with research suggesting lower-resource households feel they have fewer diet choices because of their constrained resources (40). A food-secure family may be able to choose foods according to taste preferences, whereas a food-insecure family may have fewer options when shopping on a limited budget. The lack of association between cost and consumption in this study was unexpected; other studies found that cost strongly influenced food decisions among low-resource populations (12,18), and food-insecure respondents in this study were 3 times as likely as food-secure respondents to report cost as a barrier to healthy food consumption. However, another study found low-resource households did not always report cost as a direct barrier to healthy food intake (41). Households may be so accustomed to budgeting for small amounts of fruits and vegetables that they no longer consider cost a barrier. Despite concern about food costs, food-insecure respondents in our study possibly had already budgeted for their normal rates of fruit and vegetable consumption. Food-insecure households struggling to make ends meet may also not differentiate between high costs of healthy foods and high costs of foods in general. Our sample reported consuming only 1.9 servings of fruit and vegetables daily on average. Among a population with such low fruit and vegetable consumption, costs of healthy food may not make an appreciable difference.

The lack of association between taste and cost with diets in food-insecure households may also mean the survey did not ask the right questions about barriers to healthy food consumption or that respondents conceptualized “healthy foods” differently than we did (for example, as foods like tofu or low-fat cheese). Perception of food cost among resource-limited households can include other factors besides money, including transportation, limited shopping time, and risk of food perishing (40). More research may be needed on the association between taste and cost among food-insecure populations.

Contrary to the moderating effect played by food security in taste and cost analyses, feeling too busy to prepare healthy foods (“busyness”) was associated with reduced consumption among both food-secure and food-insecure respondents. This finding is consistent with those of other studies that found busyness and preparation time to be associated with consumption, particularly among low-resource families (42,43). Given that higher rates of in-home food preparation are associated with higher diet quality (44), approaches aiming to reduce healthy food preparation time (actual or perceived), increase healthy food convenience, or provide more meal-planning resources may be appropriate for both food-secure and food-insecure populations.

**Mezzobarriers.** Mezzobarriers were not associated with healthy food consumption for either food-secure or food-insecure respondents. These findings suggest variation in perceived access in the sample, despite the study’s sampling method, which likely limited objective variation in access. However, in contrast to other studies that found perceived access to be associated with consumption rates (17,45,46), our study found no significant relationships.

This study has several limitations. First, because of the study’s cross-sectional design, only associations can be measured and no conclusions on cause can be reached. Second, the study used the terms “healthy foods” and “fruits and vegetables” interchangeably. Although people may typically think of fruits and vegetables when considering healthy foods (43) respondents in our study may have considered other food products when they responded to questions on barriers to healthy food access. Third, our study used a 26-item dietary screener to collect data on diet and calculated the outcome variable by summing reported consumption. This process does not provide data on fruit and vegetable consumption per calorie of total consumption as data collected from a full food-frequency questionnaire or a 24-hour dietary recall would provide. Fourth, the study did not have access to data on several key variables, such as household size, employment status, or budget priorities. These variables may be unmeasured confounders on the relationships of interest. Finally, the sample size and response rates were low. Respondents may not have been representative of the larger population in these Oakland neighborhoods. Future research should use larger sample sizes. 

This study contributes to our understanding of food decisions among food-insecure and food-secure households living in economically deprived neighborhoods and found that food security status modified the relationship between barriers and reported consumption. These results suggest that food security status should be included in studies investigating barriers to healthy food consumption, in addition to income, and food security status should be considered when designing and targeting dietary interventions and policies.
